# Financing HIV Programming: How Much Should Low- And Middle-Income Countries and their Donors Pay?

**DOI:** 10.1371/journal.pone.0067565

**Published:** 2013-07-05

**Authors:** Omar Galárraga, Veronika J. Wirtz, Yared Santa-Ana-Tellez, Eline L. Korenromp

**Affiliations:** 1 Department of Health Services, Policy and Practice; Brown University, School of Public Health, Providence, Rhode Island, United States of America; 2 National Institute of Public Health (INSP)/Mexican School of Public Health, Cuernavaca, Mexico; 3 Center for Global Health and Development, Boston University, Boston, Massachusetts, United States of America; 4 WHO Collaborating Centre for Pharmaceutical Policy and Regulation, Utrecht Institute for Pharmaceutical Sciences (UIPS), Utrecht, The Netherlands; 5 Department of Public Health, Erasmus MC, University Medical Center Rotterdam, The Netherlands; Indiana University and Moi University, United States of America

## Abstract

Global HIV control funding falls short of need. To maximize health outcomes, it is critical that national governments sustain reasonable commitments, and that international donor assistance be distributed according to country needs and funding gaps. We develop a country classification framework in terms of actual versus expected national domestic funding, considering resource needs and donor financing. With UNAIDS and World Bank data, we examine domestic and donor HIV program funding in relation to need in 84 low- and middle-income countries. We estimate expected domestic contributions per person living with HIV (PLWH) as a function of per capita income, relative size of the health sector, and per capita foreign debt service. Countries are categorized according to levels of actual versus expected domestic contributions, and resource gap. Compared to national resource needs (UNAIDS Investment Framework), we identify imbalances among countries in actual versus expected domestic and donor contributions: 17 countries, with relatively high HIV prevalence and GNI per capita, have domestic funding below expected (median per PLWH $143 and $376, respectively), yet total available funding including from donors would exceed the need ($368 and $305, respectively) if domestic contribution equaled expected. Conversely, 27 countries have actual domestic funding above the expected (medians $294 and $149) but total (domestic+donor) funding does not meet estimated need ($685 and $1,173). Across the 84 countries, in 2009, estimated resource need totaled $10.3 billion, actual domestic contributions $5.1 billion and actual donor contributions $3.7 billion. If domestic contributions would increase to the expected level in countries where the actual was below expected, total domestic contributions would increase to $7.4 billion, turning a funding gap of $1.5 billion into a surplus of $0.8 billion. Even with imperfect funding and resource-need data, the proposed country classification could help improve coherence and efficiency in domestic and international allocations.

## Introduction

An unprecedented increase in global spending on HIV/AIDS has occurred in the last decade; reaching over USD$16.8 billion in 2011 [1]. However, available funding continues to fall short of the global need to achieve global targets (such as the Millennium Development Goals); and after the rise in international funding slowed down due to the global crisis over 2009–12, it is now essentially flat or decreasing [2]–[4]. Better mechanisms are needed to enhance efficiency and optimize HIV allocations, on both donor and recipient sides, in relation to disease burden, as well as marginalized and vulnerable groups [1], [5]–[7].

One way to ensure sustainable funding for HIV would be through the definition of donor obligations as a set annual amount or proportion in relation to country income level. The United Nations General Assembly Special Session (UNGASS) declaration of 2001 proposed a “target of 0.7% of their gross national product for overall official development assistance, and (…) earmarking of 0.15% to 0.20% of gross national product as official development assistance for least developed countries [8].” In 2002 activists from the Global AIDS Alliance argued that the Global Fund to fight AIDS, Tuberculosis and Malaria (Global Fund) should have a similar definition of donor funding obligations as that of the UN budget; and that G-7 nations should contribute three-fourths of total requirements [9]. A so-called “equitable contributions framework” was proposed by Oxfam, *Médecins Sans Frontières* (MSF) and Fund the Fund, by which the total amount of requests made by recipient countries to the Global Fund should be divided over the 37 or 47 wealthiest countries in proportion to their GDP [10], [11]. Wealthier countries fell short on the 0.7% GDP commitment recommended by UNGASS [12]. Also, over recent years some donor nations have sometimes fallen short of, or delayed, the disbursement of their pledges to multilateral agencies such as the Global Fund. Since most health aid is short-term and volatile, it threatens the consolidation of long-term interventions; bilateral efforts, such as the United States Government’s President’s Emergency Plan for AIDS Relief (PEPFAR), generally allocate funds with an important geopolitical motivation [13]. Some multilateral institutions use formal, transparent criteria for countries’ eligibility to access funding, notably, the Global Fund has recently reformed its country eligibility criteria as well as the prioritization of funding partly due to demand surpassing available funding, and in response to criticism by donors to better align grant allocations with country program need and budget gaps [14].

Another way to increase sustainability would be to set and agree norms for domestic government HIV contributions to national HIV responses. In 2012, UNAIDS reported that domestic contributions had reached US$8.6 billion in 2011, an all-time high, yet low- and middle-income countries are encouraged to do more [1]. In 2001 the UNGASS declaration encouraged “commitments of African Heads of State or Government, at the Abuja Special Summit in April 2001, particularly their pledge to set a target of allocating at least 15% of their annual national budgets for the improvement of the health sector to help address the HIV/AIDS epidemic” [8]. External health aid may, perversely, incentivize recipient LMIC to reduce domestic contributions, and instead strategically shift government funding to the non-health sectors [15], [16] – this ‘fungibility’ is at odds with the aid additionality principle endorsed by the Global Fund and other major HIV donors. At present, there exist no clear conceptual guidelines or an appropriate frame of reference defining reasonable expected domestic contribution (EDC).

We present a new approach to defining countries’ expected domestic HIV funding contributions, redistributing actual global domestic HIV funding across low- and middle-income countries based on national income, current health spending, debt service and other relevant health economic characteristics. This modified “peer approach” [17] is appealing because it takes into account criteria and country characteristics that are relevant from a normative point of view (e.g., fairness), as well as the empirical reality of actual country epidemiologic and income contexts. Synthesizing estimates of actual domestic and donor HIV funding, expected domestic HIV funding and total resource need, we then assess each country’s HIV funding situation in relation to its epidemiologic and socio-economic situation, and discuss options for improving global and national HIV resource allocations.

## Methods

### Data Sources

Data on gross national income (GNI), income group classification as of 2010 [18], national debt service, health expenditures, and population sizes of 84 low- and middle-income countries were obtained from the World Bank [19]. Debt service are the payments on total long-term debt (public and publicly guaranteed and private nonguaranteed), the use of credit from the International Monetary Fund, and the interest on short-term debt. Long-term debt service payments are the sum of principal repayments and interest payments in the year specified. These data are collected by the World Bank Debtor Reporting System [19].We used UNAIDS estimates of national HIV incidence, actual domestic HIV/AIDS funding and external donor HIV/AIDS funding, for the most recent year available in each country (2009 or 2010), based on countries’ two-yearly progress reporting in the context of the United Nations General Assembly Special Session on HIV/AIDS (UNGASS) [1], [2], [20]–[22](Table 1).

**Table 1:** HIV program funding needs, contributions and determinants, for 84 low- and middle-income countries in US$ per person living with HIV (PLWH), unless indicated: median (and inter-quartile range) across countries.

*Variable*	*Year*	*Source*	*All*	*Quadrant I*	*Quadrant II*	*Quadrant III*	*Quadrant IV*
***Countries (N)***			84	27	16	17	24
HIV prevalence (15–49 years)	2009	UNAIDS [20]	**0.43%** (0.13−1.26)	**0.26%** (0.09−0.63)	**0.40%** (0.21−0.55)	**1.10%** (0.59−2.2)	**0.28%** (0.11−2.0)
GNI per capita (Atlas Method)	MRY	World Bank [19]	**1,915** (723−4,425)	**1,870** (570−3,675)	**1,765** (935−6,120)	**3,880** (1,170−4,500)	**1,430** (518−3,313)
Total annual health expenditures per capita	MRY	World Bank [19]	**227** (89−502)	**152** (71−501)	**279** (115–865)	**355** (126–465)	**124** (82−312)
Health expenditure pc/GNI pc	MRY	World Bank [19]	**12%** (9−15)	**12%** (10−14)	**13%** (10−17)	**10%** (8−15)	**12%** (9−16)
Debt service per capita	MRY	World Bank [19]	**142** (40−556)	**105** (20−545)	**151** (78−611)	**296** (33−511)	**91** (47−402)
Domestic annual HIV funding per PLWH:	MRY	UNAIDS [20]	**190** (32−591)	**294** (109−652)	**541** (141−932)	**143** (32−271)	**48** (10−241)
as % of total HIV funding			39%	43%	72%	39%	10%
as % of resource need			33%	25%	88%	47%	11%
as % of expected contribution			112%	197%	354%	38%	42%
Annual HIV funding per PLWH	Investment Framework [23]		**491** (236−900)	**685** (435−1,086)	**752** (604−1,304)	**368** (267−461)	**483** (129−562)
Annual HIV resource need per PLWH:	Investment Framework [23]		**570** (290−1,179)	**1,173** (565−2,271)	**617** (462−1,051)	**305 (**225−511)	**434** (251−971)
ART resource need per PLWH	Investment Framework [23]		**124** (88−240)	**119** (77−1,400)	**214** (138−364)	**124** (70−214)	**116** (90−152)
Non-ART resource need per PLWH	Investment Framework [23]		**308** (134−1,013)	**944** (281−1,977)	**320** (205−833)	**161** (121−253)	**302** (105−804)
***Countries in quadrant that are PEPFAR-co-funded (N, %)***			**11** (13%)	**2** (7%)	**3** (19%)	**5** (29%)	**1** (4%)
Global Fund HIV disbursements per PLWH	2002–10 average [46]		**85** (35−201)	**122** (49−286)	**120** (22−278)	**58** (38−235)	**59** (31−113)
***Countries in quadrant, by region, N (% of countries in quadrant or total):***							
Americas			**19** (23%)	**5** (19%)	**8** (50%)	**3** (18%)	**3 (**13%)
East Asia & Pacific			**10** (12%)	**3** (11%)	**2** (13%)	**4** (24%)	**1 (**4%)
Europe & Central Asia			**12** (14%)	**8** (30%)	**1** (6%)	**2** (12%)	**1 (**4%)
Middle East & North Africa			**6** (7%)	**1** (4%)	**0** (0%)	**0** (0%)	**5 (**21%)
South Asia			**4** (5%)	**0** (0%)	**1** (6%)	**0** (0%)	**3 (**13%)
Sub-Saharan Africa			**33** (39%)	**10** (37%)	**4** (25%)	**8** (47%)	**11 (**46%)
***Estimated Variables:***							
Expected HIV domestic contribution	2009	Regression	**169** (42–443)	**149** (29–364)	**153** (66–593)	**376** (78–484)	**114** (38–339)
Estimated Gap: Resource need minus donorscontribution minus expected domestic contribution	2009	Calculation	**45** (−112−294)	**314** (95−838)	−**164** (−453− −77)	−**221** (−326− −81)	**173** (68−304)

ART is antiretroviral treatment. GNI is gross national income. MRY: most recent year is 2009, 2008 or 2007. PEPFAR is the US President’s Emergency Plan for AIDS Relief. PLWH is person living with HIV/AIDS; pc is per capita. Resource needs per prevalent case for ART and non-ART do not exactly add to total HIV Resource Need, because these are medians, not means.

Of PEPFAR’s 15 initial focus countries, Guyana, Ethiopia, Haiti and Tanzania were not included in our analysis because of insufficient data on predictor variables. For Egypt, the resource need estimate used excludes an implausibly high HIV counseling and testing (HCT) cost line item in the UNAIDS Investment Framework, which would imply a total national resource need of over $1.4 billion, to instead assume a more plausible $39.4 million total need. For the estimated gap, we used the maximum between expected domestic contribution and actual domestic contribution within each country.

We obtained estimated national resource needs for HIV/AIDS programming from the 2011 UNAIDS Investment Framework [23] (Table 1). The Investment Framework provides national cost estimates for a comprehensive package of HIV/AIDS prevention, care and supportive services for 139 countries; but detailed costing for ART was included for only 22 high-prevalence countries. To obtain the full national resource need for the 117 countries without detailed ART costing, we imputed the missing ART costs by applying a fixed cost per-patient year of $ 1065 for low-income countries and $ 1,200 for middle-income countries, based on costs estimated among the 22 high-prevalence countries, to the national number of patients on ART in year 2010 of each of the 117 countries [21].

### Expected Domestic Contribution

We estimated expected domestic HIV contribution (EDC) per prevalent HIV case through median regression, using as predictor variables: GNI per capita, health spending per capita as a proportion of GNI per capita, and debt service per capita. The rationale for including these predictor variables, and their expected effect on domestic HIV funding, was as follows. GNI represents country income, which is expected to increase the capacity for domestic HIV funding. Health spending per capita as a proportion of GNI per capita is a proxy of the size of the health sector in the national economy, expecting that greater total spending on health would be associated with a greater capacity for domestic HIV funding. Debt service per capita represents countries’ access to credit markets, which may predict greater capacity to borrow and invest in HIV programming. For debt service per capita, 10 countries had missing values; these were imputed as the median value from the remaining 74 countries.

We chose quantile regression at the 50^th^ percentile, or “median regression” as the preferred statistical model, to minimize the influence of outliers. Median regression estimated the expected median HIV domestic contribution per person living with HIV (PLWH), conditional on the values of all co-variables. This differs from least-squares regression, which estimates the mean of the dependent variable, and is thus more sensitive to extreme values [24]. Regressions were performed in Stata 12.1, using the command *qreg* for quantile regression. (Other regression methods were used in sensitivity analyses).

We compared the resulting expected domestic contribution against the actual domestic contribution to estimate domestic contribution deviations as follows:
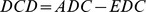
where DCD is the Domestic HIV Contribution Deviation, ADC is the Actual HIV Domestic Contribution, and EDC is the Expected HIV Domestic Contribution. We also estimated a relative measure of domestic contribution deviations as follows:



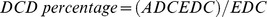



### Classifying Countries by Actual Resources Versus Resource Needs

Using the expected domestic contribution per PLWH and the UNAIDS estimates of actual domestic contribution, actual *donor* contribution and national resource needs, we calculated each country’s HIV resource gap, that is, whether the sum of *EDC* and donor contribution is greater or smaller than the overall HIV resource needs of the country:
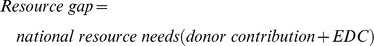



An exception was made for countries where the actual domestic contribution exceeded the *EDC:* in those cases, we estimated resource gap using the actual domestic contribution instead of the EDC, so that overall national resource gaps reflected the adequacy of donor support independent of the adequacy of domestic funding. Based on actual funding, EDC and corresponding resource gap, we then classified each country in one of the following four quadrants:

I. Resource gap exists, as resource need exceeds funding available, even though the actual domestic contribution is above the EDC.

II. No resource gap, as available domestic plus donor funding exceeds the country’s resource need, and actual domestic contributions are above the EDC.

III. No resource gap, as available domestic plus donor funding exceeds the country’s resource need, but actual domestic contribution is less than the EDC.

IV. Resource gap exists, as need exceeds funding available, and the actual domestic contribution is less than the EDC.

Finally, we summed across countries to obtain global resource needs estimates, actual and expected contributions as well as resource gaps to examine the aggregate picture.

## Results

### Actual vs. Expected Domestic Contributions

Expected domestic HIV funding contribution was estimated for 84 countries that had data available for all relevant predictor variables. The best-fitting median regression model was:
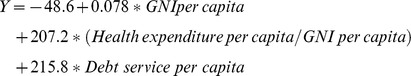
where *Y* is the domestic funding contribution for HIV/AIDS per person living with HIV, *GNI* is Gross National Income per capita. This model explained 35% of variation (R^2^ = 0.354) in actual domestic contributions across countries.

**Figure 1:**
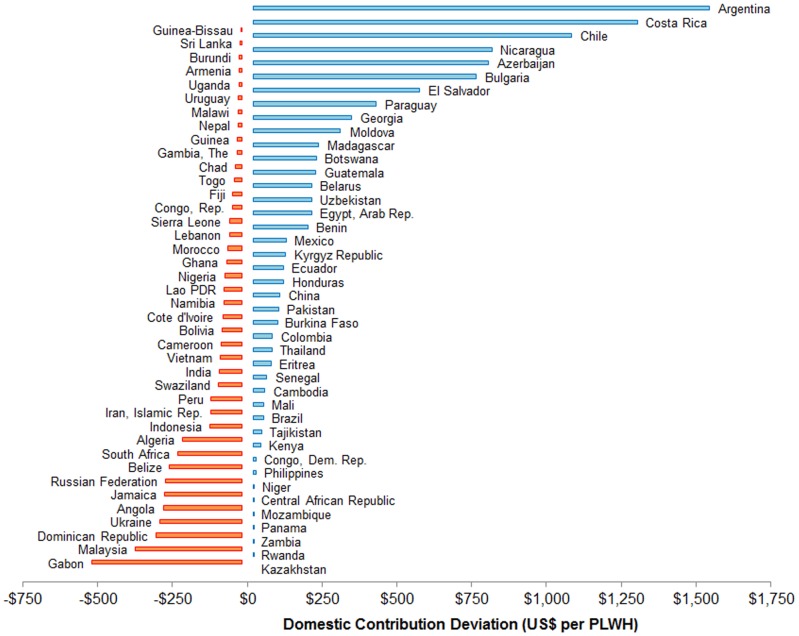
Domestic HIV Contribution Deviation: Actual minus expected domestic contributions in 2009 (in US$ per person living with HIV). Bars to the right (blue in color) represent positive deviation: Actual domestic contribution>EDC. Bars to the left (orange in color) are negative deviations: Actual domestic contribution<EDC. Deviations for Romania and Mongolia are fall off the positive scale of this graph, but are included in analyses and Table 1.

Figure 1 shows the deviations in domestic HIV contributions, defined as actual minus expected domestic contributions for 2009. Actual domestic contribution exceeded EDC in 43 countries (blue bars); but it was below EDC in 41 countries (orange bars). The largest shortages of ADC relative to EDC were estimated for Gabon ($499 shortage per PLWH), Malaysia ($355), Dominican Republic ($287), Ukraine ($272) and Angola ($261). On the other extreme, the highest excess domestic contributions relative to EDC were found for Romania ($3,830 per PLWH), Mongolia ($2,188), Argentina ($1,525), Costa Rica ($1,286), and Chile ($1,065). In general, the magnitude of both shortages and excesses in actual domestic contribution relative to EDC increased with countries income level, as expected, since both actual ADC and EDC per PLWH increase with income level.

**Figure 2:**
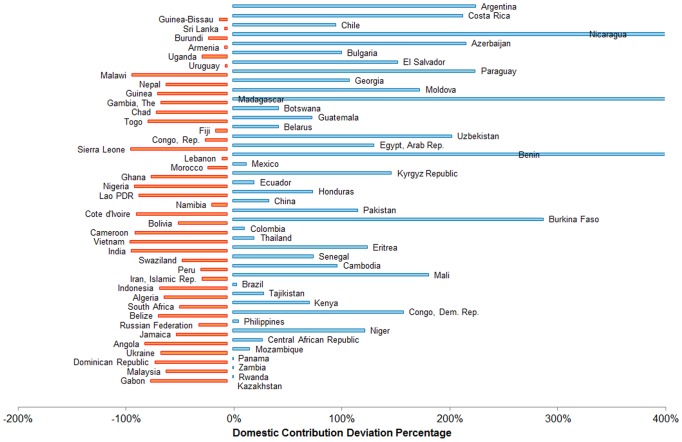
Relative Domestic HIV Contribution Deviation: (Actual – EDC)/EDC. The figure presents the relative deviation from the expected domestic contribution (EDC). Bars to the right (blue in color) are positive deviations while bars to the left (orange in color) are negative deviations. The values for Mongolia (1739%), Nicaragua (695%), and Madagascar (2876%) fall off the scale, but are included in the analyses and Table 1.

Figure 2 shows the corresponding relative deviations in domestic HIV contributions. For example, Bulgaria (101%) is contributing domestically about double its EDC. The highest positive deviations were estimated for Benin (549%), Burkina Faso (288%), Madagascar (2876%), Mongolia (1739%), Nicaragua (695%), and Romania (325%) with large relative positive deviations. On the other hand, Malawi (−88%), Ghana (−70%), Nigeria (−86%), India (−89%) and Vietnam (−90%) had large relative negative deviations.

### Domestic and Donor Contributions Relative to Resource Needs

**Figure 3:**
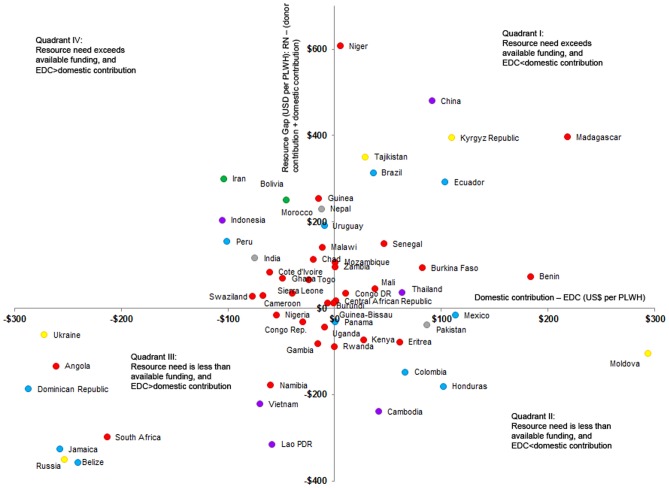
National HIV control resource availability and gaps, and the balance between domestic and donor funding contributions, in 2009. The x-axis is the national domestic contribution per person living with HIV (PLWH) in US$ minus the expected domestic contribution (EDC) per PLWH in US$; the y-axis is the HIV/AIDS resource gap: resource need minus international donor funding and minus actual domestic contribution, each per PLWH in US$. The color of the circle refers to region: red for Africa, purple for East Asia/Pacific, gray for South Asia, green for Middle East, yellow for Europe and Central Asia, and light blue for the Americas. Outliers not shown on graph (but included in analyses and Table 1) are: in Quadrant I (Argentina, Azerbaijan, Bulgaria, Egypt, El Salvador, Paraguay, Philippines, Georgia, Belarus, Kazakhstan, Romania, Uzbekistan); Quadrant II (Botswana, Costa Rica, Chile, Nicaragua, Mongolia); Quadrant III (Fiji, Gabon, Malaysia); and Quadrant IV (Algeria, Armenia, Lebanon, Sri Lanka).

Figure 3 shows the countries’ classification according to actual domestic and donor funding relative to resource need. Relative to resource need (of an overall median of $570 per PLWH), 51 countries have a gap in HIV funding, of a median $314 for 27 countries in Quadrant I, and $173 for 24 countries in Quadrant IV (Table 1). In contrast, 33 other countries have an ‘excess’ of HIV funding, of a median $164 in Quadrant II and $221 in Quadrant III. Across all 84 countries, the median funding gap is $45 per PLWH.

Countries falling in Quadrant I include Romania, Argentina, Azerbaijan, Bulgaria, Benin and Burkina Faso. Quadrant I includes comparatively more middle-income countries with relatively low HIV prevalence (median GNI per capita of $1870, adult HIV prevalence of 0.26%) and high resource need of $1173 per PLWH. Despite domestic funding above the expected, total funding available in these countries falls short of the need. These countries and their donors should examine efficiency of spending in view of the high resource need per PLWH, and donors may consider increasing funding mobilization as actual domestic contribution already exceeds EDC.

Countries in Quadrant II, which have sufficient (or more than sufficient) funding from both domestic and donor sources, include Mongolia, Nicaragua, Botswana, Cambodia and Chile. Quadrant II also covers low-HIV-prevalence countries (median prevalence of 0.40%) that have high domestic HIV funding per PLWH (median of $541) and also high total health spending per capita ($279). Total annual HIV funding per prevalent case for Quadrant II is $752, much higher than the median $368 and $483 in Quadrants III and IV). Given the more-than-sufficient domestic and donor contributions, countries in Quadrant II may have reason to scrutinize their technical efficiency in HIV funding utilization.

Quadrant III covers countries which would have more than sufficient total funding if their domestic contribution would be as expected: Dominican Republic, Nigeria, South Africa, Ukraine and Vietnam. These countries tend to have a high GNI (median of $3,880) and high HIV prevalence (1.1%) compared to countries in other quadrants. In addition Quadrant III has the largest proportion of PEPFAR focus countries (29%: 5 out of 17 countries including the high-HIV-prevalence Namibia and South Africa), compared to Quadrants I (7%), II (19%) and IV (4%; Table 1).

Countries in Quadrant IV have insufficient funding from both domestic sources and donors, and include Kazakhstan, Armenia, Lebanon, Algeria, Bolivia, Swaziland and Sri Lanka. Overall health spending per capita (median of $124), HIV spending per prevalent case ($48) and debt service per capita ($91) are lower than in countries of other quadrants.

**Figure 4:**
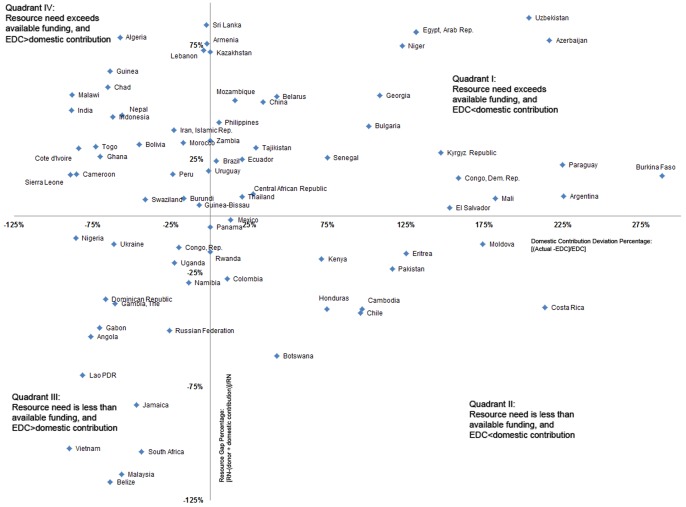
Relative Domestic Contribution Deviation and Funding Gap. The x-axis presents the domestic contribution deviation in relative terms, as percentage of the expected domestic contribution (EDC); while the y-axis shows the relative resource gap as a percentage of resource need. The following outliers fall off the scale: Benin (549%, 13%), Fiji (−11%, −220%), Guatemala (74%, −130%), Madagascar (2876%, 44%), Mongolia (1739%, −314%), Nicaragua (695%, −69%) and Romania (325%, 14%). All countries are included in the analyses and in Table 1.

When expressing domestic and total funding gap or excess relative to national resource needs (Figure 4), five countries in Quadrant III would have more than double of what they need in resources if their actual domestic contribution was at least equal to EDC: Fiji, Belize, Malaysia, South Africa, and Vietnam. For South Africa (y = −104%), our model predicts a funding excess even though current domestic funding is less than half of expected (x = −44%). Similarly, for Russia our model suggests that there is 50% more HIV funding than needed (y = −50%) though current domestic HIV funding contribution is only a third of expected (x = −26%). On the other extreme, in Quadrant I, Argentina has a funding gap of 9% of its needs (y = 9%), even though it is itself funding 225% of its expected contribution (x = 225%).

Aggregating across the 84 countries, for all PLWH in 2009 total estimated resource need totaled $10.3 billion, actual domestic contributions $5.1 billion and actual donor contributions $3.7 billion. If domestic contributions were raised to the expected level in countries where the actual was below the expected domestic contribution (Quadrants III and IV), total domestic contributions would increase to $7.4 billion, turning the funding gap for the 84 countries of $1.5 billion into a surplus of available funding of $0.8 billion.

### Sensitivity Analyses

We explored several alternative regression models to estimate expected domestic contribution, to assess the robustness of results obtained with the base-case median regression presented above. Sensitivity results are presented in terms of the number of countries that switch quadrant, and the number of countries with quadrant switches across the diagonal (i.e., from Quadrant I to III, or from II to IV, or vice versa). Diagonal switching is important because it represents a major change in the potential policy recommendations. The first sensitivity analysis added a quadratic term for GNI per capita, since the effect of country income on capacity for domestic HIV funding may be non-linear. This added variable was however not statistically significant, and it contributed very little predictive power to the overall model (R^2^ = 0.356 instead of the base-case 0.354; Table 2).

**Table 2:** Sensitivity analyses of country classification by funding situation, using alternative regression models to define expected domestic contributions and alternative imputation of missing ART resource needs.

		Number of countries in:					
Base-case and sensitivity analyses	R^2^ for actual vs. expected domestic contribution	Quadrant I	Quadrant II	Quadrant III	Quadrant IV	Countriesswitchingquadrant	Countries switching from quadrants I to III, III to I, II to IV, or IV to II
Base-case model predicting actual domestic contribution from domestic contribution (Figures 1–4) using median regression	0.354	27	16	17	24	n/a	n/a
Adding GNI per capita squared	0.356	30	20	12	22	13	0
Robust regression	0.394	22	17	19	26	12	0
Least squares regression	0.457	20	12	26	26	19	1
Impute ART resource needs missing from UNAIDS Investment Framework as $792(instead of $1,065) for low-income countries, and $1,454(instead of $1,200) for middle-income countries (based on: [27]).	Same as base-case	26	17	19	22	5	0

Second, we added a dummy variable for country income group, to account for income-related effects over and above per-capita GNI (e.g., if income group influences countries’ eligibility or priority for donor funding or international development assistance [25]). This model did not qualitatively change the main results (not shown). Similarly, a robust regression([26], *rreg* in Stata) including 82 countries (excluding 2 outliers) showed similar results to the base-case median regression, with coefficients for the four predictor variables all in the same direction and of similar magnitude. Finally, a least squares results gave coefficient values of same direction and magnitude, with an adjusted R^2^ of 0.46. With robust regression, a total of 13 countries switched quadrants, but none switched diagonally. With least squares regression ([26], *reg* in Stata), 19 countries switched quadrants but only one switched diagonally.

A last sensitivity analysis focused on ART-specific resource need, which comprises about a fifth (22%) of total resource need, ranging across quadrants from 10% in Quadrant I to 41% in Quadrant III. Since ART resource need for 117 countries was imputed from the UNAIDS Investment Framework estimates from 22 other countries, in this sensitivity analysis, we changed the imputed ART resource need (for countries without a detailed costing in the UNAIDS Investment Framework) to be $792 per person-year (instead of base-case $1,065) for low-income countries, and $1,454 (instead of base-case $1,200) for middle-income countries – using median person-year cost estimates from a recent systematic review [27]. This variation in cost assumptions changed quadrant classification for 5 countries : Burundi (IV → III), Central African Republic (I → II), Guinea Bissau (IV → III), Mali (I → II), and Panama (II → I).

In summary, the sensitivity analyses suggest that the country quadrant categorizations seem to be robust against alternative specifications of expected domestic contributions, and the critical assumptions regarding imputed values.

## Discussion

We present a simple model situating countries in terms of resource need relative to actual current domestic and donor funding for HIV programming. Our analysis identified some remarkable imbalances among low- and middle-income countries, with the large variation among countries in domestic and donor funding for HIV not always explained by differences in need. The imbalances may relate to the current debate in the development field featuring two broad camps. One advocating for more foreign aid [28], and the other proposing that countries should “pull themselves up by their bootstraps” [29]. There is also a perception that there are “basket cases” and “donor darlings”, meaning that some countries have more difficulty in making progress because of a complex relation of historical, political and economic characteristics, while others are more effective in transforming foreign aid into tangible (even if slow) progress and thus are the preferred recipients for donors [30].

Several countries with domestic contributions above the expected are from Latin America (five out of 27 in Quadrant I, and eight out of 16 in Quadrant II) including Ecuador, Mexico, Brazil, Colombia, Honduras and Guatemala. High spending on antiretroviral treatment (ART) per person in these countries may reflect higher costs per PLWH than other regions [27], [31], [32]. The higher costs may reflect higher salaries, or higher prevailing prices of antiretroviral drugs due to difficulty or unwillingness to negotiate with large pharmaceutical companies [33], [34]. As illustrated in the sensitivity analysis, the results seem to be robust in regard to the base-case national resource need estimates, which imputed missing ART costs as a fixed $1,200 per patient-year of treatment, based on the average resource need estimates. Quadrant I also has more countries from Europe and Central Asia (eight out of 27 countries in Quadrant I) such as Belarus, Bulgaria, Georgia, Uzbekistan and Azerbaijan, possibly for the same reason.

Quadrant III is made up of countries with higher HIV prevalence, higher GNI per capita, higher debt service, higher expected domestic contribution, and the highest proportion of PEPFAR focus countries, despite a low total HIV resource need per PLWH. This quadrant is of particular interest because it begs the question: Do these countries make a relatively low domestic contribution as a (perverse) reaction to more than sufficient donor funding? [16], [35]. Indeed, Quadrant III covers many (five out of 17 countries in Quadrant III) of the focus countries of the largest global bilateral donor, PEPFAR, which had until 2009 focused its support in 15 high-HIV countries. Of 11 PEPFAR focus countries included in this analysis, available funding exceeded resource need in eight (Quadrants II and III). In contrast, disbursements from the Global Fund, the largest international HIV donor, appear to be more need-based, with average HIV disbursements per PLWH lower for Quadrant III than the other three quadrants.

This situation analysis is relevant to eligibility and counterpart financing criteria used by international donors such as the Global Fund. From 2012 onwards, the Global Fund requires all supported countries to make a minimum domestic government co-funding contribution to the disease program relative to the Global Fund’s budget for the disease program, of a proportion increasing with country income (5% for low-income countries; 35% for lower-middle-income countries, and 65% for upper-middle-income countries). From the vision that supported programs should strive to become self-sustaining over time, the proportion of domestic contributions is required to increase over the years of each grant. In addition, the Global Fund’s 2011 policy stipulates that in any year, at least 55% of funding approved for grant renewals across the portfolio should be for low-income countries, whereas upper-middle income countries that are part of the G-20 and have less than an extreme disease burden are no longer eligible for grant renewals. Future further refinements of the Global Fund’s eligibility and counterpart financing policy might build on our presented model, which considers HIV disease burden and country income level as continuous rather than step-wise (categorical) determinants of expected domestic contributions, and in addition accounts for national health expenditures and debt, as determinant variables [14], [36]. Our model resembles the counterpart financing policy of the Global Alliance for Vaccination and Immunization (GAVI) that helps poor countries introduce and scale-up child immunization programs. Since 2011, GAVI considers national income level and fiscal space to determine domestic co-financing, and the desired path to self-sufficiency [37]. Expanding fiscal space refers to opportunities for broadening the tax base, improving tax administration, obtaining grants for health programming, reprioritizing expenditures, improving efficiency, and temporarily borrowing. The WHO Commission on Macroeconomics and Health suggested that low-income countries could raise general tax revenues for health in the order of 2% of GDP [38]. The private sector may be another partner to more fully engage particularly in high-HIV, upper-middle-income countries, and in countries with large, multinational enterprises such as South Africa, Namibia and Botswana [39].

Globally if 41 countries with domestic contributions below the expected increased their domestic contributions to the expected level (Quadrants III and IV), the funding situation for the 84 countries analyzed would change from a $1.5 billion shortage into a $0.8 billion surplus of available funding. Further, if donors re-allocated aid (according to need to eliminate the funding gap, or proportionally across gap countries) from countries with ‘excess’ total funding to countries with a gap persisting after the expected domestic contribution, there will be fewer countries with a funding gap, among the 51 countries (Quadrants I and IV) that we estimated to have a funding gap even with domestic contributions equaling or above the expected domestic contribution.

Total amounts of domestic and donor funding, and the equity of donor allocations across countries are just the basic components determining the allocation efficiency. A next step would be to analyze countries’ allocations within national HIV/AIDS budgets, such as how much is spent on prevention and how much on treatment. UNAIDS has previously highlighted that countries with a concentrated epidemic, such as in Latin America and South-East Asia, should allocate a larger share of HIV funding to prevention, in particular for most-at-risk-populations [40], [41].

### Limitations

Limitations of the analysis relate in the first place to data availability and quality. Data are weak and of varying quality especially for actual national HIV funding; for example, domestic expenditures may often miss expenditures covered under general health services (such as for health workers in generalized, non-HIV specialized clinics, who deliver decentralized ART and pre-ART care). Also, governments may not precisely know program outlays and corresponding funding from all donors. Notably bilateral donors that channel funds directly to implementing agencies rather than through any government agency, such as PEPFAR, are not completely reported in countries’ expenditure reporting to UNGASS. New standards are needed for reporting of global health financial data of improving quality, transparency, and completeness [15]. As a result, our analysis excluded 55 low- and middle-income countries included in the UNAIDS’ Investment Framework. Among those excluded were four PEPFAR focus countries (Ethiopia, Guyana, Haiti and Zimbabwe), and three additional countries with a generalized HIV epidemic (Djibouti, Lesotho and Tanzania). All the 55 excluded countries, together, accounted for 11% of PLWH globally in 2009, and for $2.3 billion HIV resource need, or 18% of the HIV resource need in 2009 for all low- and middle-income countries included in the UNAIDS Investment Framework, not analyzed here [23]. Of the top-20 countries with highest *HIV prevalence* in 2009, four (Guyana, Lesotho, Tanzania and Zimbabwe) were excluded from our analysis due to missing data. Similarly, of the top-20 countries with the *highest number of PLWH,* three (Ethiopia, Tanzania and Zimbabwe) were excluded due to missing data.

Second, national resource need estimates were partially based on extrapolations from globally averaged per-patient ART costs, rather than from country-specific information. Our source, the UNAIDS 2011 Investment Framework, was in the first place meant as a global assessment, that may not precisely take into account all relevant local factors such as sizes of most-at-risk populations, regional cost variations associated with transport and health and social system infrastructures. However, a sensitivity analysis showed that quadrant classification was robust against varying ART cost assumptions within a range of independent estimates.

The data limitations may explain why, apart from the larger proportion of PEPFAR support in Quadrant III countries, and the clustering of Latin America and Eastern Europe/Central Asian countries in Quadrant I, other variables failed to predict countries’ quadrant classification, with inter-quartile ranges overlapping among the quadrants for most variables.

Third, we estimated the expected domestic contribution by a hypothetical re-distribution among countries of actually available domestic funding. This approach does not address the question of whether domestic funding may be systematically too low (or too high) across countries worldwide. This modified ‘peer approach’ takes the global status quo as given, comparing countries to their peers while adjusting for relevant health financing predictor variables. Also, EDC represents a mix of two normative variables – GDP per capita and Health expenditure per capita – and the more descriptive variable Debt service per capita – which was positively associated with actual domestic HIV funding, but cannot necessarily be taken as a normative justification for requiring higher EDC. Although the proposed method helps to describe patterns of foreign aid and international development assistance, it does not directly explain individual country results, notably why certain countries have actual domestic contributions above what is expected (Quadrants I and II). Further analysis is required to understand resource allocation at country level. Above-expected contributions may result from pronounced and successful HIV/AIDS advocacy, and/or from unique ‘matching funds’ provided by international donors such as the Global Fund, who require their funding to be additional instead of replacing domestic contributions, possibly causing a relative over-prioritization of HIV/AIDS compared to other health and development needs including health systems strengthening [42], [43]. It will useful to analyze in more detail how vertical programming may be both a cause and a consequence of current global funding patterns.

The regression estimating expected domestic contributions identified two outliers with extremely high actual domestic spending compared to the expected domestic spending per PLWH: Mongolia and Romania. In Romania, this was due to high actual domestic spending in 2009 (and 2008), on the HIV response overall and on ART– provided for free in the public sector – in particular. The high program cost per PLWH was also evident from high 2010 and 2011 ART budgets reported in Romania’s UNGASS report 2012 [44]. Romania’s high domestic expenditure on ART may (in part) be explained by inefficient (centralized) ARV procurement [44]. In Mongolia, high spending per PLWH related firstly to a low number of PLWH. Mongolia’s estimated incidence of new HIV infections is high compared to prevalent HIV infections, which justifies a relatively high spending on prevention activities. Mongolia’s UNGASS 2012 report shows that prevention was almost 50% of expenditure while treatment was about 10% [45]. Because we chose median regression as the model to estimate expected domestic contributions, these outliers did however not inappropriately influence estimated EDC for the remaining countries.

### Conclusions

This study proposes a general framework to identify resource allocation imbalances between national domestic and donor contributions, and between domestic plus donor contributions and national resource needs. The resulting country classification points to which countries may deserve more or less domestic and donor allocations, based on best data available, but along with a strong plea to improve these data and the methods to estimate national resource needs and gaps. The marked imbalances among countries in actual and expected domestic and total funding relative to need suggest considerable opportunities for improved efficiency within countries and globally. Recipient and donor countries need find creative ways to improve how they transform scarce inputs into more and better-quality health outcomes. The imbalances also reflect the larger challenge in donor health funding, which is often determined by political considerations and the feasibility of exercising funds, rather than solely by real country need. The algorithm proposed, we believe, is an example of how it may be possible to identify specific disparities in both domestic and international HIV/AIDS funding, with a view towards making this funding more rational and efficient so as to maximize the impact of HIV prevention and treatment programming.

## References

[1] UNAIDS (2012) Together We Will End AIDS. In: UNAIDS, editor. Geneva: Joint United Nations Program on HIV/AIDS (UNAIDS); World Health Organization (WHO).

[2] UNAIDS (2010) Joint Action for Results: UNAIDS Outcome Framework 2009–2011. Geneva: The Joint United Nations Program on HIV/AIDS (UNAIDS).

[3] Kaiser Family Foundation (2012) Financing the Response to AIDS in Low- and Middle-Income Countries: International Assistance from Donor Governments in 2011. Washington, DC: Kaiser Family Foundation.

[4] Leach-Kemon K, Chou DP, Schneider MT, Tardif A, Dieleman JL, et al (2012) The global financial crisis has led to a slowdown in growth of funding to improve health in many developing countries. Health Aff (Millwood) 31: 228–235. 22174301 10.1377/hlthaff.2011.1154

[5] Kerouedan D (2010) [The Global Fund to fight HIV/AIDS, TB and Malaria 5-y: evaluation policy issues]. Bull Soc Pathol Exot 103: 119–122. 20376595 10.1007/s13149-010-0051-2

[6] Global Fund (2010) Value for money improving value for money in Global Fund-supported programs Geneva: The Global Fund to Fight AIDS, Tuberculosis and Malaria.

[7] Amico P, Gobet B, Avila-Figueroa C, Aran C, De Lay P (2012) Pattern and levels of spending allocated to HIV prevention programs in low- and middle-income countries. BMC Public Health 12: 221. 22436141 10.1186/1471-2458-12-221 PMC3328263

[8] United Nations General Assembly (2001) Agenda item 8. In: Nations U, editor. United Nations General Assembly, Twenty-sixth special session (UNGASS). New York: United Nations.

[9] Kapp C (2002) Global Fund faces uncertain future as cash runs low. Lancet 360: 1225. 12401252 10.1016/S0140-6736(02)11294-3

[10] Burton B (2004) Australia's contribution to global health fund provokes dismay. Bmj 328: 486. 10.1136/bmj.328.7438.486 PMC351837 14988175

[11] Zeitz PS (2003) What is the US Fair Share of Funding Needed to Catalyze an expanded and Comprehensive Response to the Global AIDS Crisis?. In: Global AIDS Alliance, editor. International Committee, Presidential Advisory Council on HIV/AIDS. Bethesda MD.

[12] Brugha R (2005) The Global Fund at three years–flying in crowded air space. Trop Med Int Health 10: 623–626. 15960700 10.1111/j.1365-3156.2005.01437.x

[13] Lane C, Glassman A (2007) Bigger and better? Scaling up and innovation in health aid. Health Aff (Millwood) 26: 935–948. 17630435 10.1377/hlthaff.26.4.935

[14] Global Fund (2011) Policy on eligibility criteria, counterpart financing requirements, and prioritization of proposals for funding from the Global Fund.. Geneva: The Global Fund to Fight AIDS, Tuberculosis and Malaria.

[15] Lu C, Schneider MT, Gubbins P, Leach-Kemon K, Jamison D, et al (2010) Public financing of health in developing countries: a cross-national systematic analysis. Lancet 375: 1375–1387. 20381856 10.1016/S0140-6736(10)60233-4

[16] Garg CC, Evans DB, Dmytraczenko T, Izazola-Licea JA, Tangcharoensathien V, et al (2012) Study raises questions about measurement of 'additionality,'or maintaining domestic health spending amid foreign donations. Health Aff (Millwood) 31: 417–425. 22323173 10.1377/hlthaff.2008.0815

[17] Savedoff WD (2007) What should a country spend on health care? Health Aff (Millwood) 26: 962–970. 17630438 10.1377/hlthaff.26.4.962

[18] World Bank (2010) Country Classification Data. Washington, D.C. : World Bank.

[19] World Bank (2011) Data catalog. Washington, D.C.: World Bank.

[20] UNAIDS (2012) Country Reported Data for 2009 for 84 Countries.

[21] WHO UNAIDS, UNICEF (2011) Global HIV/AIDS response: Epidemic update and health sector progress towards Universal Access. Progress report 2011. Geneva: World Health Organization (WHO).

[22] UNAIDS (2010) The 2010 UNAIDS report on the global AIDS epidemic. Geneva: The Joint United Nations Program on HIV/AIDS (UNAIDS).

[23] Schwartlander B, Stover J, Hallett T, Atun R, Avila C, et al (2011) Towards an improved investment approach for an effective response to HIV/AIDS. Lancet 377: 2031–2041. 21641026 10.1016/S0140-6736(11)60702-2

[24] Koenker R (2005) Quantile regression. Cambridge; New York: Cambridge University Press. xv, 349 p. p.

[25] Lordan G, Tang KK, Carmignani F (2011) Has HIV/AIDS displaced other health funding priorities? Evidence from a new dataset of development aid for health. Soc Sci Med 73: 351–355; discussion 356–358. 10.1016/j.socscimed.2011.05.045 21745705

[26] Andersen R (2008) Modern methods for robust regression. Los Angeles: Sage Publications. xv, 107 p. p.

[27] Galárraga O, Wirtz VJ, Figueroa-Lara A, Santa-Ana-Tellez Y, Coulibaly I, et al (2011) Unit costs for delivery of antiretroviral treatment and prevention of mother-to-child transmission of HIV: a systematic review for low- and middle-income countries. Pharmacoeconomics 29: 579–599. 21671687 10.2165/11586120-000000000-00000 PMC3833352

[28] Sachs J (2005) The end of poverty : economic possibilities for our time. New York: Penguin Press. xviii, 396 p. p.

[29] Easterly W (2006) The white man's burden : why the West's efforts to aid the rest have done so much ill and so little good. Oxford; New York: Oxford University Press. 380 p. p.

[30] Collier P (2007) The bottom billion : why the poorest countries are failing and what can be done about it. Oxford; New York: Oxford University Press. xiii, 205 p. p.

[31] Kaplan WA, Ritz LS, Vitello M, Wirtz VJ (2012) Policies to promote use of generic medicines in low and middle income countries: A review of published literature, 2000–2010. Health Policy. 10.1016/j.healthpol.2012.04.015 22694970

[32] Wirtz VJ, Santa-Ana-Tellez Y, Trout CH, Kaplan WA (2012) Allocating scarce financial resources for HIV treatment: benchmarking prices of antiretroviral medicines in Latin America. Health Policy Plan. 10.1093/heapol/czs011 22367770

[33] Shadlen KC (2012) The Mexican Exception: Patents and Innovation Policy in a Non-conformist and Reluctant Middle Income Country. European Journal of Development Research 24: 300–318.

[34] Shadlen KC (2009) The Politics of Patents and Drugs in Brazil and Mexico: The Industrial Bases of Health Policies. Comparative Politics 42: 41-+.

[35] Bokhari FA, Gai Y, Gottret P (2007) Government health expenditures and health outcomes. Health Econ 16: 257–273. 17001737 10.1002/hec.1157

[36] Global Fund (2011) Decision Points of the Twenty-Fifth Board Meeting Accra, Ghana, 21–22 November 2011– Decision Point GF/B25/DP16: Modification of Grant Renewals and Transition to New Funding.. Twenty-Fifth Board Meeting Accra, Ghana, 21–22 November 2011: The Global Fund to fight AIDS Tuberculosis and Malaria.

[37] Saxenian H, Cornejo S, Thorien K, Hecht R, Schwalbe N (2011) An analysis of how the GAVI alliance and low- and middle-income countries can share costs of new vaccines. Health Aff (Millwood) 30: 1122–1133. 21653966 10.1377/hlthaff.2011.0332

[38] WHO Commission on Macroeconomics and Health (2001) Macroeconomics and health: Investing in health for economic development World Health Organization ed. Geneva.

[39] Thirumurthy H, Galarraga O, Larson B, Rosen S (2012) HIV Treatment Produces Economic Returns Through Increased Work And Education, And Warrants Continued US Support. Health Aff (Millwood) 31: 1470–1477. 22778336 10.1377/hlthaff.2012.0217 PMC3728427

[40] Hesterl V, McGreevey B, Hecht R, Avila-Figueroa C, Gaillard E (2009) Assessing costing and prioritization in National AIDS Strategic Plans. Working paper no. 26. Washington DC: Results for Development Institute.

[41] United Nations Economic and Social Commission for Asia and the Pacific (2008) HIV and AIDS in Asia and the Pacific: A review of progress towards Universal Access. Discussion paper prepared for the UNESCAP/UNAIDS Expert Group Meeting. Bangkok: United Nations ESCAP.

[42] Munderi P, Grosskurth H, Droti B, Ross DA (2012) What are the essential components of HIV treatment and care services in low and middle-income countries: an overview by settings and levels of the health system? Aids 26 Suppl 2S97–S103. 23303438 10.1097/QAD.0b013e32835bdde6

[43] Cohen RL, Li Y, Giese R, Mancuso JD (2012) An Evaluation of PEPFAR's Effect on Health Systems Strengthening in Sub-Saharan Africa. J Acquir Immune Defic Syndr. 10.1097/QAI.0b013e3182816a86 23254150

[44] Romania (2012) UNGASS Country Progress Report. UNAIDS.

[45] Mongolia (2012) UNGASS Country Progress Report. UNAIDS.

[46] Global Fund (2012) Approved Grant Amounts and Disbursements. Geneva, Switzerland: The Global Fund to Fight AIDS, Tuberculosis and Malaria.

